# Photoemission sources and beam blankers for ultrafast electron microscopy

**DOI:** 10.1063/1.5117058

**Published:** 2019-09-27

**Authors:** Lixin Zhang, Jacob P. Hoogenboom, Ben Cook, Pieter Kruit

**Affiliations:** 1 Department of Imaging Physics, Delft University of Technology, Lorentzweg 1, 2628CJ Delft, The Netherlands; 2 School of Instrumentation and Optoelectronic Engineering, Beihang University, 100191 Beijing, China

## Abstract

Observing atomic motions as they occur is the dream goal of ultrafast electron microscopy (UEM). Great progress has been made so far thanks to the efforts of many scientists in developing the photoemission sources and beam blankers needed to create short pulses of electrons for the UEM experiments. While details on these setups have typically been reported, a systematic overview of methods used to obtain a pulsed beam and a comparison of relevant source parameters have not yet been conducted. In this report, we outline the basic requirements and parameters that are important for UEM. Different types of imaging modes in UEM are analyzed and summarized. After reviewing and analyzing the different kinds of photoemission sources and beam blankers that have been reported in the literature, we estimate the reduced brightness for all the photoemission sources reviewed and compare this to the brightness in the continuous and blanked beams. As for the problem of pulse broadening caused by the repulsive forces between electrons, four main methods available to mitigate the dispersion are summarized. We anticipate that the analysis and conclusions provided in this manuscript will be instructive for designing an UEM setup and could thus push the further development of UEM.

## I. INTRODUCTION

Many processes such as phase transitions, chemical reactions, electron transport, and molecular vibrations occur at ultrafast time scales. These time scales can typically be as short as a few picoseconds or even femtoseconds.^1^ The development of ultrafast electron microscopy (UEM) has led to the prospect that dynamic information can be obtained at higher spatial resolution (<1 nm), as well as better temporal resolution (<100 fs) than ever before. The technique usually involves pump-probe setups, where a femtosecond or a nanosecond laser pulse illuminates the specimen and an electron pulse probes the specimen at different delay times after the laser pump pulse. Different techniques of electron microscopy can be employed, such as real-space imaging, diffraction, and electron energy loss spectroscopy.^2–5^ Applications are being pursued in many fields varying from materials science to chemistry and electronics. One of the first applications of UEM was electron beam testing of high-speed integrated circuits,^6–10^ but there is also a lot of interest in the investigation of carrier dynamics in semiconductors.^11–15^

The performance of an ultrafast electron microscope depends heavily on the quality of the ultrashort electron pulses. Despite several reviews on the concept and applications of UEM, for example, Refs. 16–23, there are a few that cover the instrumentation involved. In this review, we look at the current state-of-the-art photoemission sources and beam blankers to deliver ultrashort electron pulses for high resolution, ultrafast imaging and diffraction, and give an outlook for the future development of the instrumentation.

This paper is divided into five parts. Basic requirements and parameters for UEM are presented in Sec. II, where we also summarize the different imaging modes of UEM. In Sec. III, we present an overview of photoemission sources and beam blankers used in UEM experiments and then estimate the reduced brightness of the photoemission sources. Conclusions are given in Sec. IV, and we make an outlook for future work in Sec. V.

## II. BASIC REQUIREMENTS AND PARAMETERS FOR UEM

### A. Modes of UEM

The most frequently used imaging and diffraction modes in UEM include stroboscopic mode (e.g., Refs. 2, 24 and 25), repeated (multiple-shot) mode (e.g., Refs. 26 and 27), and single-shot mode (e.g., Refs. 28–30). They all set different requirements to the electron pulse.

#### 1. Stroboscopic mode

In 1968, Plows and Nixon^31^ applied the stroboscopic technique in a scanning electron microscope (SEM) in order to measure the time dependent voltages in microelectronic devices. In this method, a single electron or a few electrons per pulse are used to slowly build up an image at a certain time in a reversible and repeatable process. This process can be started by a light pulse or an electronic signal. This technique is still the most widely used method in UEM, for both imaging and microdiffraction. From a theoretical point of view, identical spatial resolutions can be obtained in stroboscopic mode as compared to traditional electron microscopy because the signal from many pulses can be added. Practical considerations such as required stability may, however, limit spatial resolution.

#### 2. Repeated mode

The difference between stroboscopic and repeated modes is the number of electrons included in a pulse. For this mode, typically a bunch of 10 000–50 000 electrons^26,27^ is used. Images are either taken at one particular region on the sample or at a different sample region, assuming, of course, that the information from the different shots is essentially identical. Compared with the stroboscopic mode, the advantage of the repeated mode is the reduction in the number of pulses needed. Therefore, imaging time, sample repeatability requirements, and noise are reduced. In some instances, this mode can also be used to image a nonreversible process.^27^ The disadvantage of the repeated mode is a reduction in both spatial resolution and time resolution as compared to that of the stroboscopic mode.

#### 3. Single-shot mode

The single-shot method was pioneered by Dömer and Bostanjoglo,^32^ who realized a spatial resolution of about 200 nm at a temporal resolution of about 10 ns. For this mode, typically a bunch of 10^5^–10^8^ electrons is used. This limits the spatial resolution in the scale of tens to hundreds of nanometers. Lagrange *et al.*^33^ redesigned the electron optics in a dynamic transmission electron microscope (DTEM), improving the spatial resolution to 10 nm with a temporal resolution of 15 ns. While the temporal resolution of the single-shot imaging mode is still low compared to those of the other modes of UEM, this technique can be used to measure nonreversible processes. In Sec. III, we will see that a single-shot diffraction pattern can be obtained with picosecond or even subpicosecond resolution by using pulse compression techniques.^29,34,35^

### B. Definitions of parameters for photoemission sources

The properties of the stream of electron pulses emitted from the electron source will directly influence the quality and achievable resolution of the images. Therefore, the electron source is the vital part of the entire setup. Often, reduced brightness, emittance, or current density^36,37^ are used to make a quantitative characterization of the electron sources. In literature, one may, however, find different definitions, for instance, for electron beam brightness. Therefore, we will start by providing the formal definitions for some important experimental parameters and then use these definitions for our analyses in Secs. II C and III.

#### 1. Pulse duration

The pulse duration *τ* is one of the most important parameters in UEM, as it determines the temporal resolution of the experiments. A measure for *τ* that can be used irrespective of the shape of the temporal profile is the shortest time containing 50% of the number of electrons within a pulse (*N*). For Gaussian shaped pulses, we can directly convert other measures, such as FWHM, and standard deviation to the full width containing 50% (FW50). An advantage of using FW50 is that this parameter is less sensitive to the tails of the distribution or the occurrence of hotspots within the distribution than other measures. Since most manuscripts do not state how *τ* was measured, we assume it is the full width containing 50% (FW50) of the electrons unless otherwise stated. When a root mean square (RMS) value is given, we double it when we quote pulse length.

#### 2. Reduced/normalized emittance

The emittance ε describes how the beam evolves in the spatial/momentum domain. The two-dimensional emittance can formally be defined from the positions and transverse momenta of all electrons in the beam with respect to the center of the beam. It is also possible to define the emittance in terms of positions and the angles with respect to the optical axis. When *z* denotes the direction along the optical axis, the two-dimensional RMS emittance can be calculated for the *x*-direction as

$$\varepsilon_{x}=\sqrt{\langle x^{2}\rangle \langle \theta_{x}^{2}\rangle -\langle x\theta_{x}\rangle^{2}},$$ (1)

where ⟨⟩ indicates averaging over the entire distribution, x is the position of the electrons within the beam, and $\theta_{x}$ is the angle $v_{x}/v_{z}$, with $v_{x}$ and $v_{z}$ the electron velocity in *x*- and *z*-directions, respectively. $\varepsilon_{y}$ follows in the same way but using the *y* coordinate. This definition makes the emittance rather sensitive to outliers and is thus most useful for well-known distributions. We would prefer to use a definition for emittance which is related to the smallest ellipse in the x- and $\theta_{x}$-planes (or y- and $\theta_{y}$-planes) that contains half of the particles. This makes the value of the emittance less sensitive to the tails of the particle distribution in x- and $\theta_{x}$. In order to get an approximate equality between the definition in Eq. (1) and our “full width 50” definition, we should take only one quarter of the size of this ellipse. The exact relation between these two definitions depends on the particle distribution.

The emittance can be determined anywhere in the beam: as long as there are no stochastic Coulomb interactions in the beam, the beam is neither accelerated nor encounters an aperture; the emittance is a conserved quantity as followed from Liouville's theorem. However, there are certain planes along the trajectory, where it is easier to relate the emittance to simple properties of the beam than in other planes. For instance, in the emission plane of a source or for a focused beam, the emittance can be defined as $\varepsilon =r_{f}\theta$, where $r_{f}$ is the focus or source radius and θ is a typical half opening angle of the electrons at the focus or source. The exact value of the emittance now depends on the definition of *r_f_* and *θ*. In a focused beam which opening angle has been limited by an aperture, we would take the FW50 radius of the focus and the full half opening angle. Another example of a plane where the emittance is easily related to known parameters is when the beam is spread out over a larger area on the sample as in transmission electron microscopy: the emittance can then be defined using the radius *r_ill_* of the irradiated area instead and the half-angle of the illumination beam as seen from the sample.

To describe the beam along its trajectory in the electron microscope, it is more convenient to work with a quantity that is conserved also under acceleration and/or when passing through an aperture. Under acceleration, the angles with respect to the optical axis, and thus the emittance as defined in this paper, decrease. This effect can be compensated by adding the z-velocity into the equation. For instance, the normalized emittance is conserved under acceleration when defined as

$$\varepsilon_{n}=\beta \gamma \varepsilon ,$$ (2)

where $\beta =v_{z}/c$, $\gamma =1/\sqrt{1-\beta^{2}}$, and c is the speed of the light.

Alternatively, we can scale with acceleration energy instead of velocity, which gives us the reduced emittance: $\varepsilon_{r}=\varepsilon \sqrt{V_{r}}$, with $V_{r}=V+(\frac{eV^{2}}{2m_{0}c^{2}})$. Note that for a focused beam, the reduced emittance thus follows as:

$$\varepsilon_{r}=r_{f}\theta \sqrt{V_{r}}.$$ (3)

To work with a quantity that is also conserved when an aperture is used, we need to turn to the brightness.

#### 3. Reduced and normalized brightness

The reduced brightness is the beam characteristic that is conserved both under acceleration and when passing an aperture. We thus consider this the most important parameter for characterizing a beam in an electron microscope. The reduced brightness directly gives us the current in a probe or the current in a coherent area when practical parameters such as the radius of the illuminated or focus spot area and the half opening angle are known.

We start with the definition of the brightness which is given as follows: ^36,37^

$$B=\frac{\mathrm{dI}}{\mathrm{dAd}\Omega },$$ (4)

where *dA* is the area through which a current *dI* passes from within a solid angle *d*Ω. With this definition, different parts of the beam may have a different brightness. For a full beam, at a focus, we get an approximation to the average brightness in the beam when we take

$$B=\frac{I}{\pi r_{f}^{2}\pi \theta^{2}},$$ (5)

with *r_f_* the FW50 of the focus size and *θ* as the maximum half angle of a top-hat angular current distribution and *I* the full current in the beam. Note that we have the emittance in the denominator, and brightness can thus also be interpreted as the current within a certain emittance

$$B=\frac{I}{\pi^{2}\varepsilon_{x}\varepsilon_{y}}.$$ (6)

Now, the reduced brightness^37,38^ can be defined equivalently using the reduced emittance from Eq. (3), which gives, in a focus

$$B_{r}=\frac{I}{\pi^{2}r_{f}^{2}\theta^{2}V_{r}}.$$ (7)

When an aperture is placed in the beam at a plane where the current distribution is approximately uniform, this reduces either the area or the angle. However, in this case, the current is proportionally reduced, and so the reduced brightness remains constant after an aperture. It then also follows that, analogous to Eq. (7), *B_r_* can be defined anywhere along the beam trajectory using the local radius of the beam and the internal opening angle of the beam at that same position instead of *r_f_* and *θ*. In SI units, reduced brightness is expressed in A/(m^2^ sr V). Alternatively, a normalized brightness *B_n_* can be defined related to the normalized emittance. It can be seen that reduced and normalized brightness are related via

$$B_{n}=B_{r}m_{0}c^{2}/q=5.12\times 10^{5}B_{r},$$ (8)

where $m_{0}=9.11\times 10^{-31}\;\text{kg}$ is the rest mass of the electron, $c=3\times 10^{8}\;m/s$ is the speed of light, and $q=1.6\times 10^{-19}\;C$ is the elementary charge; so, the prefactor is equal to the rest mass of the electron in units of electron-volt.

As the reduced brightness is conserved along the beam trajectory and our aim is to compare different UEM sources, it is convenient to look at *B_r_* in terms of source properties. Substituting $\theta =v_{r}/v_{z}$, current density $J=I/\pi r^{2}$, and $\mathrm{eV}=\frac{1}{2}mv_{z}^{2}$ into Eq. (7), *B_r_* can be rewritten as

$$B_{r}=\frac{\mathrm{eJ}}{\pi E_{t}},$$ (9)

where $E_{t}=\frac{1}{2}mv_{r}^{2}$, and *v_r_* is the velocity in the radial direction. We now see that $B_{r}$ is solely determined by the current density and the transverse energy of the electrons. This is particularly useful when applied at the cathode surface and, for instance, leads to an expression for the reduced brightness of a photoemission source^39^

$$B_{r}=\frac{\mathrm{eJ}}{\pi (h\nu -\phi )},$$ (10)

where hν is the energy of the photons illuminating the source and ϕ is the work function. If we define the excess energy as the difference between the photon energy and work function, so $E_{t}=h\nu -\phi$, this is the maximum transverse energy that an electron could acquire. For a thermal emitter, such as the Schottky source, the transverse energy is kilotesla. For a cold field emitter, it is dependent on the field strength and tunneling coefficient and the work function, a typical value for *E_t_* is 1 eV.

With these expressions, we have several possibilities to estimate the brightness of a beam when other parameters are given in the description of an electron source. We want to stress that even the definitions of emittance and brightness can already give rise to different values of these parameters with possible deviations of up to a factor of 2.

### C. Requirements for imaging and diffraction

#### 1. Imaging

First, we will discuss the imaging mode of UEM. We assume a square image field where the size of one pixel is matched to half of the resolution of the instrument. Therefore, with $d_{\mathrm{res}}$ the pixel size, and $N_{\mathrm{pix}}$ the number of pixels in an image, $d_{\mathrm{res}}\sqrt{N_{\mathrm{pix}}}$ is the full width of the illumination area. Then, with *θ* the internal half angle in the illumination as seen from the sample, $\varepsilon_{\mathrm{im}}=\frac{\theta d_{\mathrm{res}}}{2}\sqrt{N_{\mathrm{pix}}}$ is the emittance of the beam forming the image. Now, with $N_{\mathrm{im}}$ the number of electrons in the image and *T* the total illumination time used to form the image, Eq. (7) for the brightness of the beam that is required to form the image within the given time with the given number of electrons can be rewritten as follows:

$$B_{r}=\frac{qN_{\mathrm{im}}}{\pi^{2}\varepsilon_{\mathrm{im}}^{2}V_{r}T}.$$ (11)

The required number of electrons per pixel depends on the number of gray values one wants to distinguish from the shot noise. Here, the conversion of impinging electrons into a detector signal also plays a role and thus, for the entire experiment, it is important to choose a camera with the highest possible detection quantum efficiency. State-of-the-art detectors can reach nearly 100% detection quantum efficiency. Thus, 100 electrons impinging per pixel, giving a noise level of 10 electrons, is what we deem the minimum number giving distinguishable gray levels. Then, for a 1 mega pixel image $N_{\text{pix}}=10^{6}$, $N_{\text{im}}=10^{8}$. If we aim to get atomic-scale resolution in the image, say $d_{\text{res}}=0.1\;\text{nm}$, multiplied by the length of the image in pixels, $\sqrt{N_{\mathrm{pix}}}$, we get $l_{\text{ill}}=100\;\text{nm}$. The required *θ* determines the partial coherence, and its value depends on the exact application and acceleration voltage. Here, we take 1 mrad, which is typical for high resolution electron microscopy (HREM) at 100 kV.^40^ A good way of expressing the requirement is to take the product of measurement time and reduced brightness

$$B_{r}T=\frac{qN_{\mathrm{im}}}{\pi^{2}\varepsilon_{\mathrm{im}}^{2}V_{r}}\approx 5\times 10^{3}\;\left[A\;{s/(m}^{2}\text{srV})\right].$$ (12)

This result is independent of the imaging mode described in Sec. II C 1. For continuous operation of a microscope with a typical brightness of 10^8^ A/(m^2^srV), this would mean that image acquisition is possible at 50 *μ*s per image. There is no camera that can keep up with this.

When we apply the equation to a single-shot image, i.e., T=τ, with N = 10^8^ and say, τ=1  ps, the instantaneous brightness should be 5 × 10^15^ A/(m^2^srV). If for repeated imaging we set the number of electrons used in each single illumination $N=10^{4}$, we need in total 10^4^ pulses to form the image with $N_{\text{im}}=10^{8}$. This would then require B_r_ = 5 × 10^11^ A/(m^2^srV) for τ=1   ps, or when using a source of brightness 10^8^ A/(m^2^srV), a minimum τ=1   ns. For better time resolution, we would have to give up on spatial resolution. In the stroboscopic mode, it is, in principle, possible to work at any number of electrons per pulse, even far below one. As a side remark, while talking about one electron per pulse, it is important to distinguish between beams with one electron per pulse at the source and beams with one electron per pulse at the sample, because in electron microscopy, the current at the sample is often orders of magnitude smaller than the current from the source. We find that in some publications, this is not very well indicated.

In scanning transmission electron microscopy (STEM), the beam is focused to a small probe. The resolution in the image is equal to the size of the probe. Since in STEM only one pixel is illuminated at a time, the beam is apertured to a smaller emittance than the beam for TEM and thus has a smaller current, fewer electrons per pulse. This means that ultrafast STEM (see Ref. 41) will require even longer acquisition times than ultrafast TEM.

#### 2. Diffraction

Similar to the derivation above, but now for a total number of electrons *N_diff_* in the diffraction pattern and a reduced emittance *ε_diff_*, we can rewrite Eq. (7) to determine how brightness affects a diffraction pattern

$$B_{r}=\frac{qN_{\mathrm{diff}}}{\pi^{2}\varepsilon_{\mathrm{diff}}^{2}V_{r}T}.$$ (13)

A diffraction pattern gives an average location of the atoms and therefore less information than an image. The information is concentrated in fewer pixels, and thus, we need fewer electrons, say $N_{\text{diff}}=10^{6}$. For example, Aeschlimann *et al.*^26^ used multishot pulses with N = 50 000 electrons and Siwick *et al.*^42^ used 150 pulses with N ≈ 6000 electrons to form a diffraction pattern.

Then, we should determine *ε_diff_*. To form a diffraction pattern, the coherence length of the electron wave $X_{\mathrm{pc}}$ must be several times the lattice spacing.^40^ Taking silicon, for example, 10 times the lattice spacing means $X_{\text{pc}}=5\;\text{nm}$. Since $X_{\mathrm{pc}}=\frac{\lambda }{2\theta }$,^37^ the reduced brightness can be written as

$$B_{r}=\left(\frac{8meX_{\mathrm{pc}}^{2}}{\pi^{2}h^{2}}\right)\frac{N_{\mathrm{diff}}q}{r_{\mathrm{ill}}^{2}T}=6.7\frac{N_{\mathrm{diff}}q}{r_{\mathrm{ill}}^{2}T}.$$ (14)

In ultrafast diffraction, most work is done on large samples with r≈100 μm, for example,^43–46^ which is called large area diffraction (LAD). Therefore, we could set $r_{\text{ill}}=100\;\mu m$. The brightness requirement is now

$$B_{r}T=6.7\frac{N_{\mathrm{diff}}q}{r_{\mathrm{ill}}^{2}}=10^{-4}\;\left[A\;{s/(m}^{2}\text{srV})\right].$$ (15)

This is very different from the imaging requirement. For instance, for single-shot diffraction with a beam of B_r_ = 10^8^ A/(m^2^srV), the time needed is $T=\tau_{\text{atomic}}=1\;\text{ps}$. However, to record the diffraction pattern in 1 ps would then require an instantaneous current of 0.16 A; so, the question is if there are sources that can combine the high brightness and high current requirements and keep all that charge together in the pulse. Repeated and stroboscopic diffractions decrease the requirements on the number of electrons per pulse.

In Table I, we list the required reduced brightness and current for different modes of imaging and diffraction for a time resolution of 100 fs, according to the discussion above. Note that imaging and diffraction also require very different values of the energy spread in the beam: for imaging, it is important to keep the relative amount of energy spread low to minimize chromatic aberrations, while diffraction can be done at 100s to 1000s eV energy spread.

**TABLE I.** Approximate reduced brightness *B_r_* required to operate in the various modes of imaging and diffraction with a time resolution of τ = 100 fs. For imaging, the spatial resolution is d_res_ = 0.1 nm with 1 megapixels per image, V_r_ = 100 kV, N_im_ = 10^8^. For diffraction, the illuminated area has a radius of 100 *μ*m and N_diff_ = 10^6^. For the repeated mode, we assume 10^4^ electrons per pulse, and for the stroboscopic mode, we assume 1 electron per pulse. Note that the total illumination times for single-shot, repeated, and stroboscopic modes then become 100 fs, 1 ns, and 10 *μ*s, respectively, for imaging, and 100 fs, 10 ps, and 100 ns for diffraction. HREM: high resolution electron microscopy. LAD: large area diffraction.

	Single shot	Repeated	Stroboscopic
*B_rHREM_* [A/(m^2^srV)]	5 × 10^16^	5 × 10^12^	5 × 10^8^
*I_HREM_* (A)	1.6 × 10^2^	1.6 × 10^–2^	1.6 × 10^–6^
*B_rLAD_* [A/(m^2^srV)]	1 × 10^9^	1 × 10^7^	1 × 10^3^
*I_LAD_* (A)	1.6	1.6 × 10^–2^	1.6 × 10^–6^

## III. METHODS TO CREATE ULTRASHORT ELECTRON PULSES

At present, there are two main methods to generate ultrashort electron pulses, which are through photoemission or by beam blanking. In this section, we review the photoemission sources and beam blankers for which we found information in the literature, and we make comparisons and comments on their properties in terms of the discussion from Sec. II.

### A. Types of photoemission sources

#### 1. Flat photoemission sources

This is the most often used type of photocathode, either illuminated from the front or from the back by a laser pulse. According to our review of photoemission sources, the back illuminated cathodes are adopted by most of the research groups for diffraction. For imaging, front- or side-illumination seems to be more often used. A variety of different flat designs may be found, ranging from uniform thin films coated on transparent substrates to the finite area LaB_6_ cathodes. The shape and width of the laser illumination profile, or the area with photoemission material, determine the area from which the electrons are emitted, and thus, there is a limit to how small this can be. For instance, for the four LaB_6_ photoemission sources in our review, emission areas ranged from 15 to 150 *μ*m in radius.^47–50^ Since the current density *J* comes directly into the equation for brightness [see Eq. (5)], a high brightness flat photocathode is also a high total current electron source. In order to limit the space charge effects, it is then necessary to accelerate the electrons as fast as possible; so, the cathode is usually in a strong field.

#### 2. Sharp tip photoemission sources

Sharp tip photocathodes started out as cold field emission sources, usually made of a tungsten wire etched with a tip radius between 1 μm and a few nanometers.^51–53^ The laser usually comes from the side and is polarized in the direction of the optical axis. The sharp tip concentrates the electric field close to the tip; so, the electrons feel a strong acceleration right after emittance. Because of the small size, the sharp tip photocathode has a much smaller emittance than a flat cathode and can be expected to reach a higher reduced brightness. Recently, Schottky emitters have also been used for photoemission.^2,54–56^ The Schottky emitter is usually a tungsten tip with a radius of about 1 *μ*m, covered with ZrO to lower the work function. The strong electric field lowers the work function even further by the Schottky effect; so in the continuous mode, the thermal emission is sufficient for a high brightness. For pulsed emission, the tip must be at a lower temperature to avoid the background signal in between the pulses, risking the effect of losing the work function effect of the ZrO. Methods to optimize the operation are still under investigation.

### B. Overview of photoemission sources for UEM

In this section, we start by reviewing as many photoemission sources as possible, based on the parameters we have discussed in Sec. II B. The result is given in Table S1 in the supplementary material; a summary of the results per source type is given in Table II. Our calculation of source parameters should be considered as estimation because we have to base ourselves on the information in the reviewed papers, which is sometimes limited. In the supplementary material, we list how we calculated the values listed for each particular source. In some cases, the information in the manuscript can lead to different values for the brightness, when information is combined in different ways. In these cases, we have presented the calculated maximum reduced brightness. Some photoemission sources designed for UEM experiments, such as Refs. 29, 57–60, are not included in Table S1 because some important parameters for calculating reduced brightness are not available in those papers.

**TABLE II.** Summary of typical parameters for each type of photoemission source obtained from our literature review (see the supplementary material Table S1 for the full list of parameters for each individual source). Schottky- and Cold Field Emission (CFE)-based cathodes denote (modified) commercial source unit, and custom sharp tip denotes the use of a home-built source. *N*: number of electrons per pulse, *τ*: pulse duration (FW50), *B_r_*: reduced brightness.

Type of sources	Main materials of the source	Radius of the source *r_source_*	Typical *N* at source plane	Typical τ	The best reported experimental τ	Typical *B_r_* A/(m^2^srV)
Flat photocathodes	Au, Ag, Cu, LaB_6_	Tens of micrometers	10^3^–10^6^	Subpicosecond to picosecond	230 fs (Ref. 61)	10^1^–10^7^
Custom sharp tip photocathodes	W (ZrO), Ta	Submicrometers to micrometers	10^0^10^2^	Subpicosecond	65 fs (Ref. 53)	10^6^–10^8^
Schottky-based photocathodes	W (ZrO)	Submicrometers	∼10^2^	subpicosecond	200 fs (Ref. 54)	10^6^–10^8^
CFE-based photocathodes	W	Tens of nanometers	∼10^1^	Subpicosecond	360 fs (Ref. 62)	∼10^9^
RF source	Cu, Mg	Tens of micrometers to millimeters	∼10^7^	10s–100s fs	100 fs (Ref. 35)	10^5^–10^7^
RF compression	Au	Tens of micrometers	10^0^–10^1^	100s fs	200 fs (Ref. 34)	∼10^8^
Ultracold plasma	…	Tens of micrometers	∼10^4^	100s ps	850 ps (Ref. 63)	∼10^5^

With respect to the calculations, there are some aspects which are noteworthy:

1) In most papers, we can find information about the beam current, source emission radius, the work function of the source, and the energy of the laser irradiated on the source. When the work function of the cathode is not given, but the energy spread of the beam is, we use the energy spread also for the maximum transverse energy. With these parameters, we can calculate the reduced brightness using Eqs. (7), (9), and/or (10);
2) sometimes, we cannot find the values for parameters at the photocathode plane, but we do obtain the current, probe size, convergence angle, etc., on the sample plane so that we can still use Eq. (7) to calculate reduced brightness. It should be noted that we cannot use mixed parameters from both the source plane and the sample plane to calculate brightness using Eq. (7);
3) if we know the normalized brightness, we can use Eq. (8) to convert it to reduced brightness;
4) one thing we should be cautious of is that usually the values for the current reported in the papers are the average current, which is *I* = *f* × *N* × *q* with *f* the frequency of the pulses. From this equation, we can calculate the number of electrons in a pulse and then calculate the current in a single pulse using *I* = *Nq*/*τ*;
5) when we use the parameters at the sample plane to calculate the brightness, we do not automatically have the number of electrons at the source plane, which is an interesting parameter to compare. However, for some sources, we know the typical emittance at the source plane, and by comparing the emittance at the sample plane to the emittance at the source plane, we can sometimes still make an estimate of the number of electrons at the source plane.
6) we often use the size of the source in our calculations. There is a physical size of the emitting surface of a cathode and there is a virtual source size. For the brightness calculation, we always take the parameters in the same plane; so, when that is the cathode plane, we combine the physical size with the transverse energy in the cathode plane. When we take the virtual source size, we combine this with the aperture angle as coming from the plane in which the virtual source is located. So, the fact that a virtual source size can be smaller than the physical size of the cathode is always accompanied by an increase of the aperture angle at the virtual source. In fact, it is the aperture angle at the cathode plane that determines the virtual source size and the size of the emitting area at the cathode that determines the angle from the virtual source.
7) in all calculations, the space charge and Coulomb interaction are not explicitly considered, but if the parameters at the sample plane are used for the calculation, this should be implicitly included.

### C. Overview of beam blankers for UEM

Electron pulses can also be created by sweeping a continuous beam at a high speed over a narrow slit. The beam is off most of the time, which is the reason that this is called a beam blanker. In Table III, we present the information about the beam blankers reported in the literature for use in UEM. In this report, we do not consider how to design a beam blanker or discuss the performance of specific beam blankers in detail.

**TABLE III.** Parameters for reviewed beam blankers. *H*: total height of the blanker; *L*: active length of the single deflector plate; d: distance between deflector plates; *V_beam_*: energy of electron beam; *V_def_*: voltage on the deflector plates; *f*: frequency of blanking signal; I: average beam current after the blanker; τ: temporal resolution; the brightness and energy spread depend on the used electron microscope. Energy spread due to the blanker has only been determined for a limited number of systems (i.e., 4, 10, 11, and 12). The number with an asterisk means that it is a theoretical or computational work.

Number (reference)	Type	*H *×* L* × *d* (mm)	*V_beam_* (kV)	*V_def_* (V)	*f* (MHz)	*I* (pA)	*τ* (ps)
1^31^	Static plates	…	…	5	7	…	100
2^69^	Static plates	d = 0.5	30	400	1	2	…
3^70^	Static plates	…	…	…	0.04	…	…
4^25,67,71^	Deflector + buncher	356.5 × 14.5 × 2	20	…	1000	10	0.2
5^72^	Plug-in beam chopping system	60 × 6 (3) × 0.3 (0.2)	3	5	250	2.5	10
6^68^	Elliptical plates	…	10	64	18 000	…	0.11
7^73–75^	Horse shoe double plate	51.8 × 11.3 × 2	10	5	160	…	1600
8^76,77^	Commercial static plates	L = 6, d = 0.3	4	10	10	0.15	90
9^66^	Microwave cavity (TM_110_)	H = 17.1	30	…	3000	…	0.1
10^78^	Microwave cavity (TM_110_)	H = 16.7	200	…	3000	2.7	1.1
11^64^,^*^	MEMS parallel plates	5 × 0.1 × 0.001	30	10	20 000	1.3	0.4
12^65^,^*^	MEMS parallel plates	L = 0.01, d = 0.001	30	10	100	0.16	0.1

Our aim is to compare the performance of beam blankers to that of photoemission sources. According to Table III, it can be seen that most of the traditional beam blankers are designed with centimeter scale dimensions. The temporal resolution is then determined by the rise time of the electric pulse and as a result limited to tens of picoseconds at best. Some concepts have started using microfabricated parallel plates,^64,65^ which may have the potential of reaching subpicosecond pulses. Presently, only blankers with an RF cavity deflector have been shown to be able to generate picosecond or shorter electron pulses.^66–68^ An important feature of pulses created with a beam blanker is that the brightness of the pulse can be approximately the same as the brightness of the continuous beam. A slight decrease in brightness can be expected because the virtual source is moving during the pulse and thus the apparent size is slightly increased in the sweeping direction.

Compared to photoemission sources, potential practical advantages of a blanker could be the easy conversion between continuous operation and pulsed operation of the microscope and the stability of the beam. For some designs of the blankers, varying the pulse duration may also be easier than in photoemission sources. On the other hand, so far, more implementation examples and a wider range of experimental beam parameters have been demonstrated for photoemission sources compared to those for beam blankers.

## IV. OBSERVATIONS FROM THE LITERATURE REVIEW

In this report, we have reviewed 43 photoemission sources (see Table II and Table S1 in the supplementary material) and 12 beam blankers (see Table III). The emphasis was on estimating the reduced brightness in the pulse, because this is the dominating parameter for obtaining a certain image quality within a reasonable acquisition time. In many of the papers that we studied, the brightness was not explicitly given and we had to calculate an estimated maximum value from other parameters that were provided. Table II summarizes the typical values reported for the different types of sources. As can be seen in Table S1, most of the sources are used to perform ultrafast diffraction experiments, while very few sources can be used to perform ultrafast imaging.

**FIG. 1.**
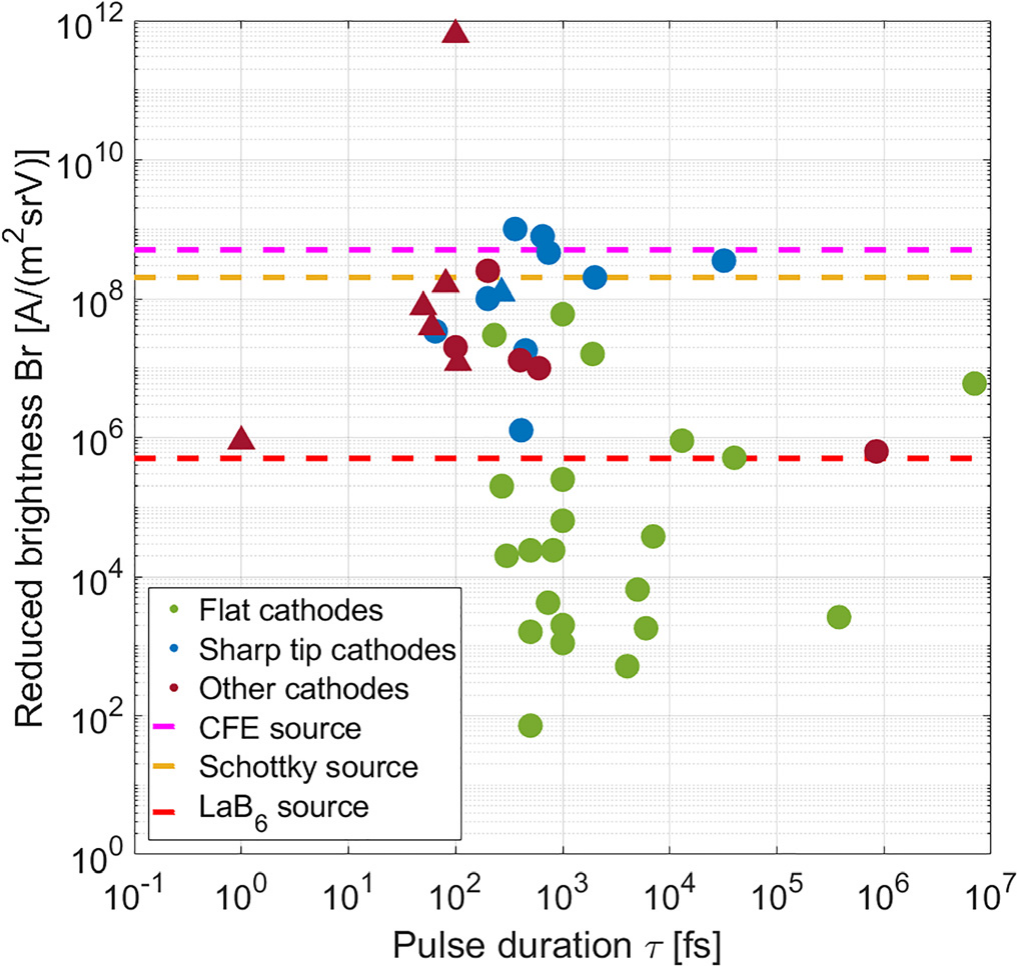
Reduced brightness calculated for each photoemission source vs the reported pulse duration. Symbol color indicates the source type and matches the color indication in Table S1 where all data points are listed. Other cathodes include sources with RF compression and acceleration. Circles indicate experimental results, and triangles represent theoretical or simulation work. Dashed lines indicate the reduced brightness for each source type in the continuous mode (cf. Table IV). As can be seen, photoemission sources can typically reach the same reduced brightness as in continuous beam operation. Pulse durations down to 200 fs are reached; shorter pulses are obtained with pulse compression and acceleration, as detailed in the main text.

For our conclusions, we focus on pulse duration and reduced brightness. In Fig. 1, we plotted both values for each of the reviewed sources. Below, we present our conclusions with respect to both characteristics.

### A. Pulse duration

The sources of numbers 1–32 are photoemission sources without additional techniques, such as radio frequency (RF) compression. As can be seen in Fig. 1, most of these sources have been used to create electron pulses with duration between 200 fs and 10 ps. Indeed, the current state-of-the-art photoemission source modified for an existing TEM can provide ∼200 fs electron pulses.^54^ Mutual repulsion of electrons in the pulse prevents the generation of sub-100 fs electron pulses; see Refs. 79–81 for a detailed estimation of temporal broadening. For shorter electron pulses, techniques like pulse compression and acceleration start to be applied; for details, see source numbers 33–43 in Table S1.

In general, there are four main methods that have been used to decrease the effects of the space charge inside the electron pulse on the temporal resolution. The first technique is to use an RF cavity integrated into the photoemission source to accelerate the electrons to relativistic energies in mega-electron-volt or even giga-electron-volt range in order to shorten the propagation time of electron pulse in the column and to effectively damp the mutual repulsion of electrons.^35,82–86^ Even the generation of subficosecond electron pulses has been theoretically suggested using this approach.^86^ However, the high voltage pulsed electron beams are difficult to use in electron microscopes and are often destructive for the materials studied, especially for biological and organic materials. Moreover, the energy spread in the pulsed beam may be of the order of kilo-electron-volt.^82,85,86^ The second method is to compress DC accelerated photoelectrons, which has been done using RF fields,^34,45,57,87^ and with an electron mirror-based pulse compressor.^88^ Third, the charge inside a pulse can be reduced to approximately one or a few electrons per pulse to avoid the space charge induced expansion.^89,90^ Finally, a compact electron source placed in close proximity to the sample can be used to shorten the propagation length,^1,18^ thus reducing the interaction time for Coulomb repulsion of the electrons in the pulse. Recently, Zhou *et al.*^91^ proposed a new concept called “adaptive electron-optical design” aiming to boost the signal-to-noise ratio while maintaining the high energy and time resolution, but it still needs to be validated in practical experiments. The time resolution of pulses from standard electrostatic beam blankers can reach sub-100 ps.^76,77^ Some new designs with miniaturized plates promise subpicosecond pulses,^64,65^ but are still under test. Also, using an RF cavity instead of the standard blankers to pulse the beam in a TEM has been shown to give very promising results for achieving subpicosecond pulses.^66,78^

### B. Reduced brightness

It is insightful to compare the highest achieved reduced brightness reported for the different types of photoemission sources to the typical values that are obtained for the sources in the continuous beam mode. These are listed in Table IV and also indicated with dashed lines in Fig. 1. As can be seen, the photoemission sources reach values for the reduced brightness that are equivalent to what can be reached in a continuous mode electron microscopy.

**TABLE IV.** Comparison of reported maximum reduced brightness for pulsed beams obtained with the different photoemission sources (see also Table II) to the typical reduced brightness that the same sources give in extraction of a continuous electron beam.

	Continuous mode *B_r_* [A/(m^2^srV)]	Pulsed modeMax. *B_r_* [A/(m^2^srV)]
LaB_6_ cathode	5 × 10^5^	10^4^
Schottky source	2 × 10^8^	10^8^
Cold field emitter	5 × 10^8^	10^9^

Flat photocathodes are the most often used type of photocathode, either illuminated from the front or from the back by a laser pulse. According to our review of the photoemission sources, the back illuminated cathodes are adopted by most of the research groups. From Tables II and S1, we can see that for most of the flat cathodes, the maximum reduced brightness is limited to ∼10^7^ A/(m^2^srV), apart from those flat cathodes with RF acceleration and/or compression techniques. Neither of those techniques is easily applicable in an electron microscope, however. Comparing these brightness values to our estimated required values in Table I, we conclude that none of these sources can perform single-shot imaging or even repeated imaging with atomic resolution. They could perform repeated large area diffraction because they usually have sufficient total current, but with picosecond timing resolution, they have most often been used for stroboscopic diffraction, in line with our results from Table I.

Sharp tip photocathodes reach brightness values of up to 10^9^ A/(m^2^srV). From our Table I, we see that this is just right for ultrafast TEM imaging with 100 fs temporal and atomic spatial resolutions with one electron per pulse at the sample.

Pulses from beam blankers (see Table III) have brightness close to the brightness of the continuous source listed in Table IV. Comparing the pulse durations in Table III with the typical values obtained with photoemission sources in Table II, we see that similar temporal resolution can be reached with beam blankers.

## V. CONCLUSIONS AND OUTLOOK

To make a “molecular movie” in which we see atoms move while a molecule changes shape, as proposed by Dwyer *et al.*^1^, is an inspiring goal for UEM. In this review, we have evaluated, to our knowledge, all photoemission sources and beam blankers used to obtain pulsed electron beams for UEM reported to date. From this evaluation, we can conclude that state-of-the-art photoemission sources using sharp tips give the same reduced brightness of about 10^9^ A/(m^2^srV) as the continuous sources. Further, we have seen that beam blankers, which already have a brightness close to that of the continuous source, are giving the same pulse durations as the photoemission sources. The reported performance of the electron sources has next been compared to the requirements that making a molecular movie imposes on the electron source. We may conclude that making a movie in the traditional sense of taking a sequence of real-space images at consecutive times is many orders of magnitude away. However, to make such a movie in the stroboscopic mode, of a molecule or a series of identical molecules going through a highly repeatable and reproducible process, is coming close. To try and use diffraction mode for this purpose does not seem to bring advantages: it still requires to collect the same amount of information from the same small area. Only when the traditional advantage of diffraction, which is averaging over a large number of similar unit cells, can be employed will this mode deliver information faster.

## SUPPLEMENTARY MATERIAL

See the supplementary material for Table S1 in supplementary material lists of the retrieved parameters for all reviewed photoemission sources. Detailed explanations on the calculations for each particular source are given, as well.
